# Supplementary material for: The GBM Tumor Microenvironment as a Modulator of Therapy Response: ADAM8 Causes Tumor Infiltration of Tams through HB-EGF/EGFR-Mediated CCL2 Expression and Overcomes TMZ Chemosensitization in Glioblastoma
Source: Cancers (Basel). 2022 Oct 7;14(19):4910. doi: 10.3390/cancers14194910 (PMC9561975; doi:10.3390/cancers14194910)
Supplement: Supplementary file 1 [file cancers-14-04910-s001.zip › Figure S5 Original Western Blot images (A Figure 1;B Figure 4; C Figure 5).pdf]

A

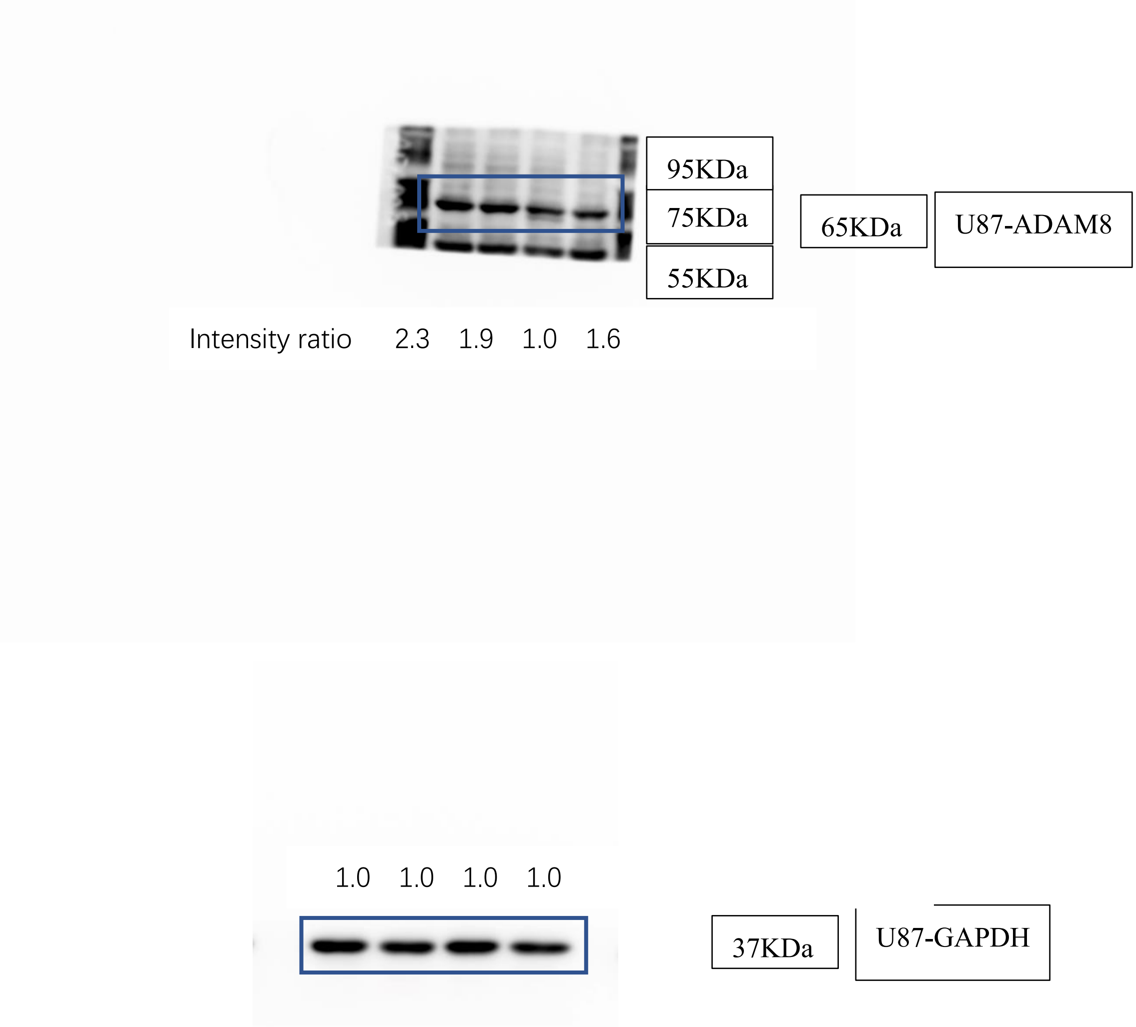

95KDa
75KDa
55KDa
65KDa
U87-ADAM8
Intensity ratio 2.3 1.9 1.0 1.6
1.0 1.0 1.0 1.0
37KDa
U87-GAPDH

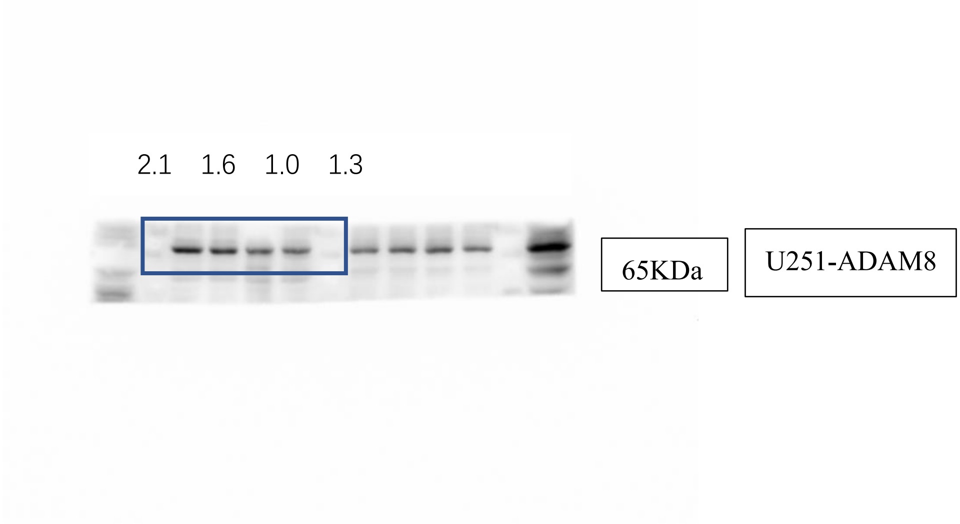
2.1 1.6 1.0 1.3
65KDa
U251-ADAM8

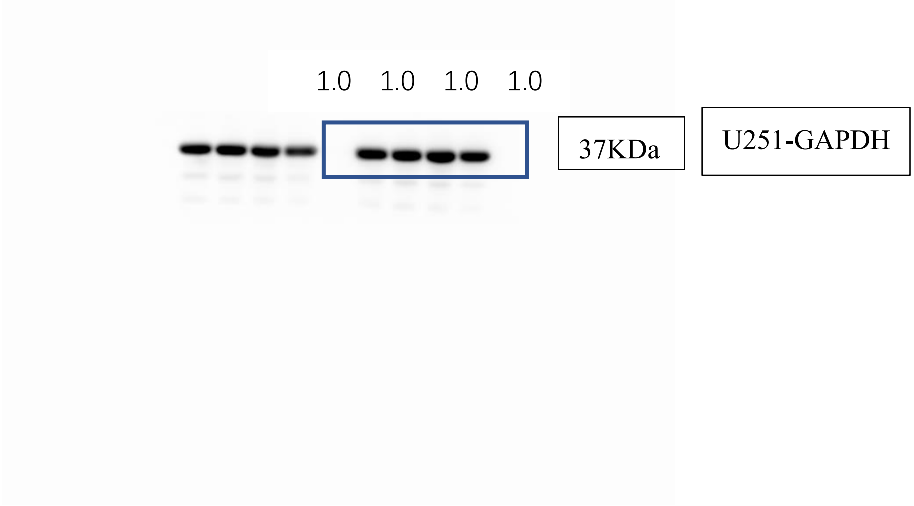
1.0 1.0 1.0 1.0
37KDa
U251-GAPDH

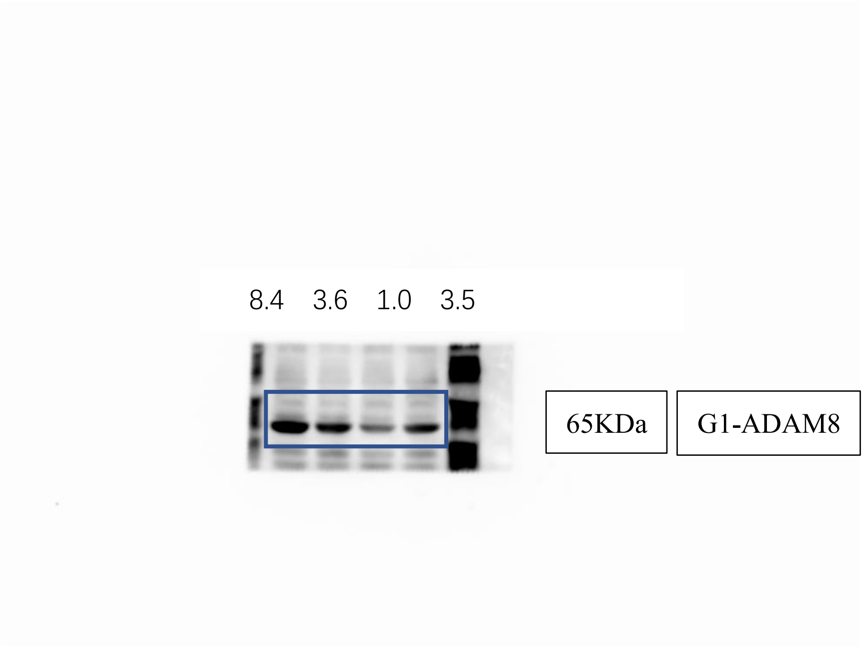
8.4 3.6 1.0 3.5
65KDa
G1-ADAM8

1.0 1.0 1.0 1.0

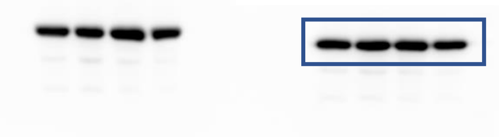

37KDa G1-GAPDH

3.5 2.6 1.0 1.4

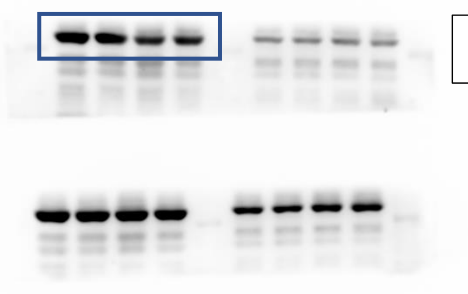

65KDa G2-ADAM8

1.0 1.0 1.0 1.0

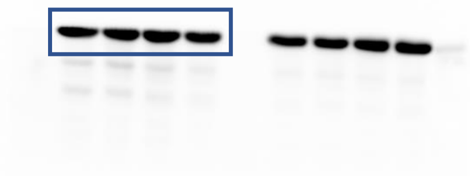

37KDa G1-GAPDH

B

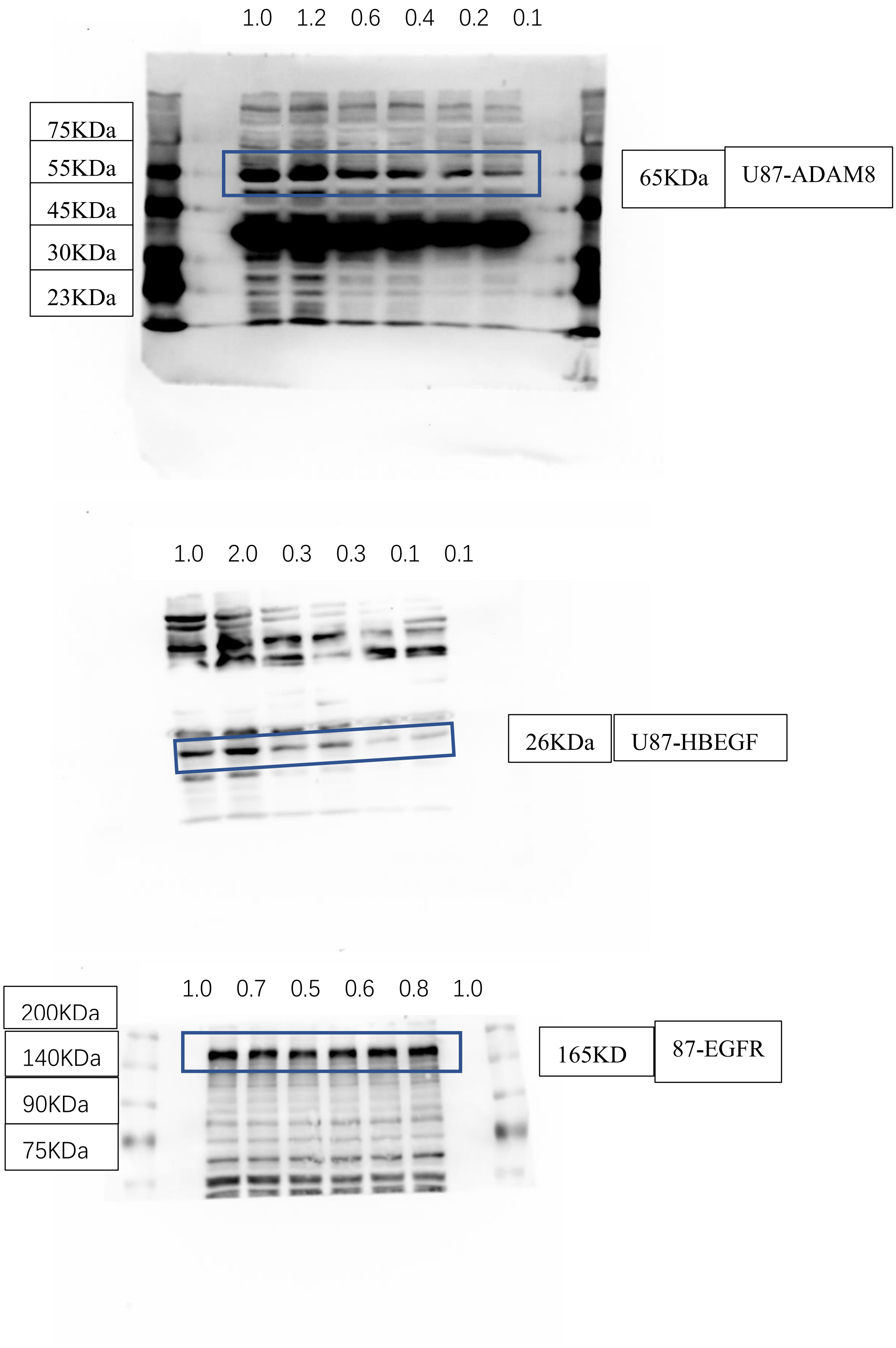
1.0 1.2 0.6 0.4 0.2 0.1
75KDa
55KDa
45KDa
30KDa
23KDa
65KDa
U87-ADAM8
1.0 2.0 0.3 0.3 0.1 0.1
26KDa
U87-HBEGF
1.0 0.7 0.5 0.6 0.8 1.0
200KDa
140KDa
90KDa
75KDa
165KD
87-EGFR

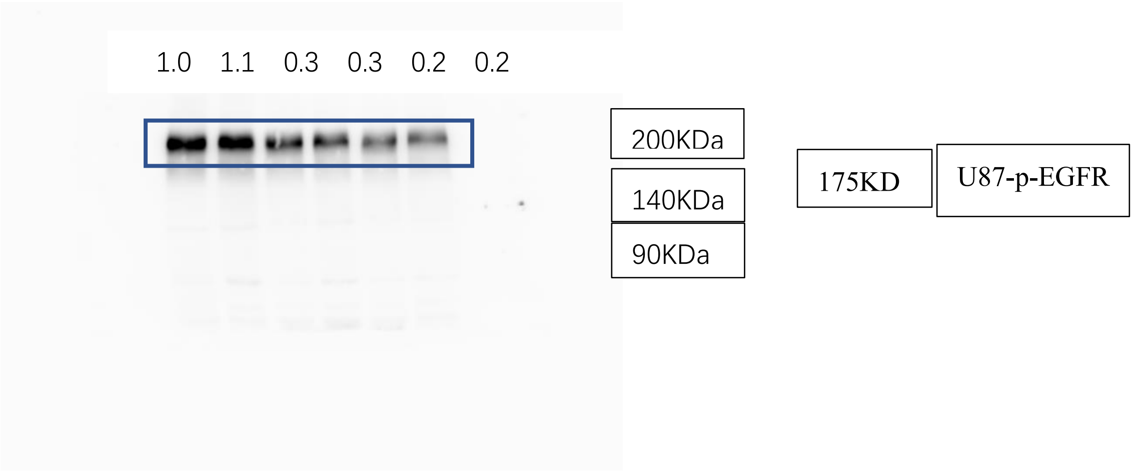
1.0 1.1 0.3 0.3 0.2 0.2
200KDa
140KDa
90KDa
175KD
U87-p-EGFR

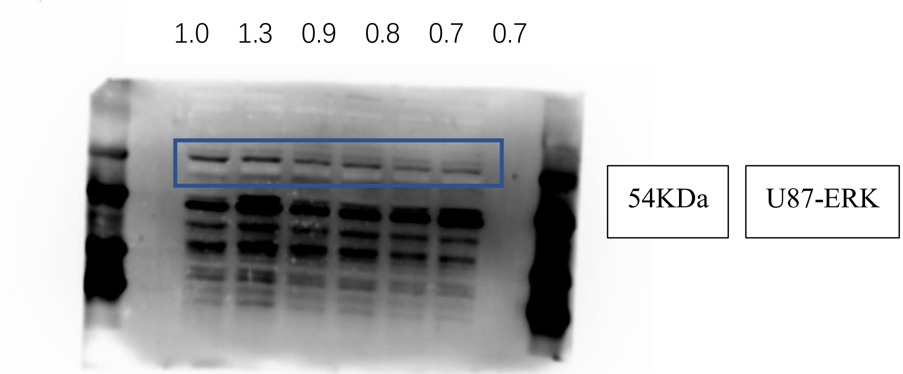
1.0 1.3 0.9 0.8 0.7 0.7
54KDa
U87-ERK

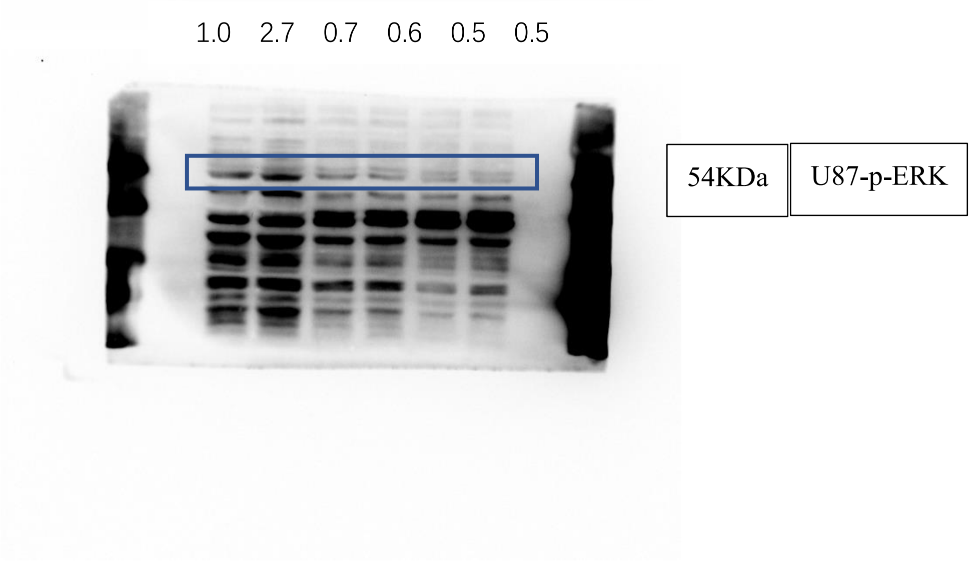
1.0 2.7 0.7 0.6 0.5 0.5
54KDa
U87-p-ERK

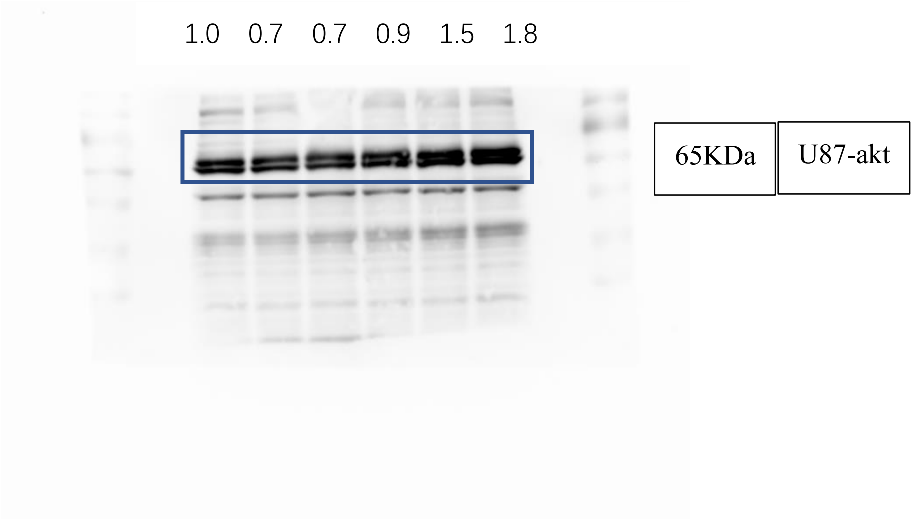
1.0 0.7 0.7 0.9 1.5 1.8
65KDa
U87-akt

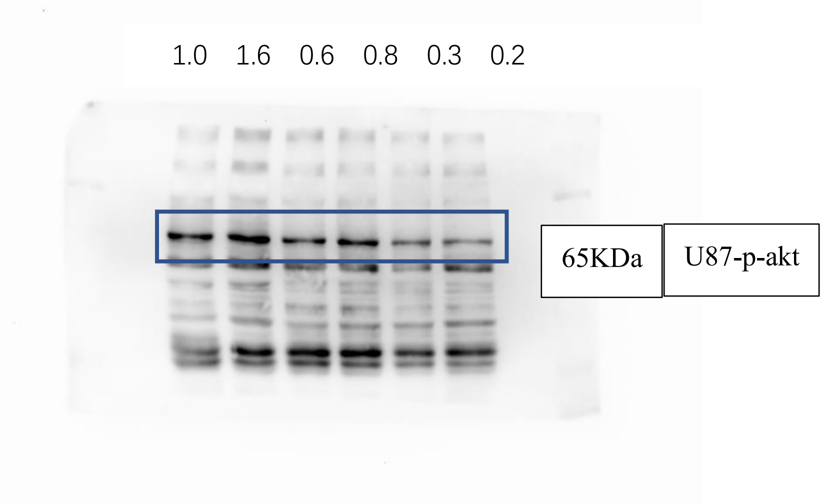
1.0 1.6 0.6 0.8 0.3 0.2
65KDa
U87-p-akt

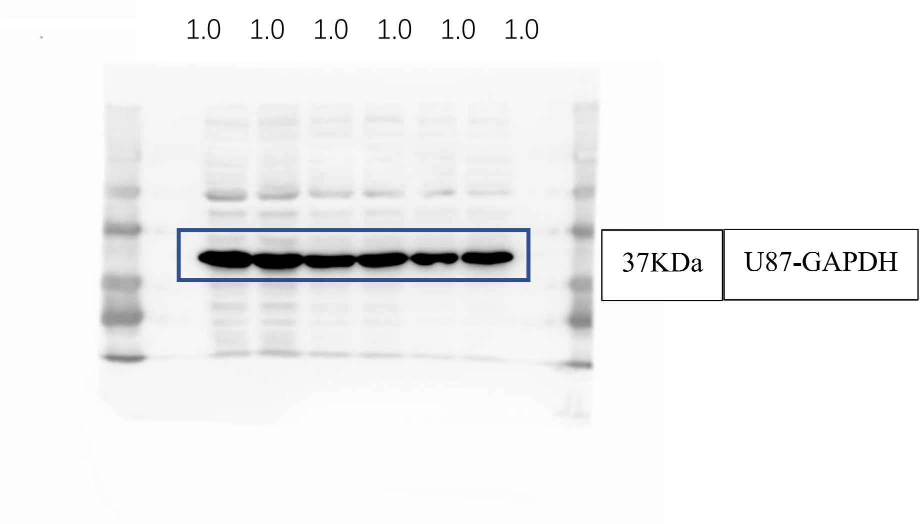
1.0 1.0 1.0 1.0 1.0 1.0
37KDa
U87-GAPDH

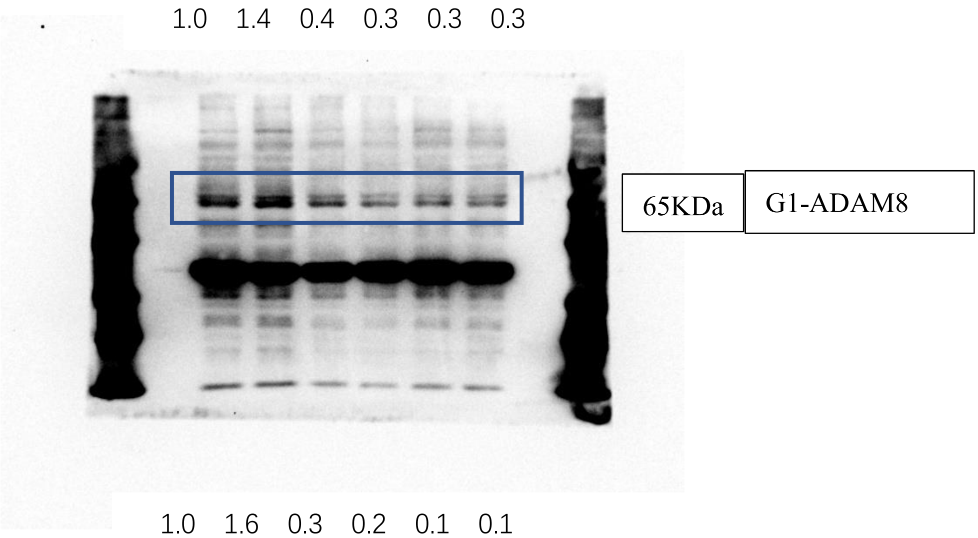
1.0 1.4 0.4 0.3 0.3 0.3
65KDa
G1-ADAM8

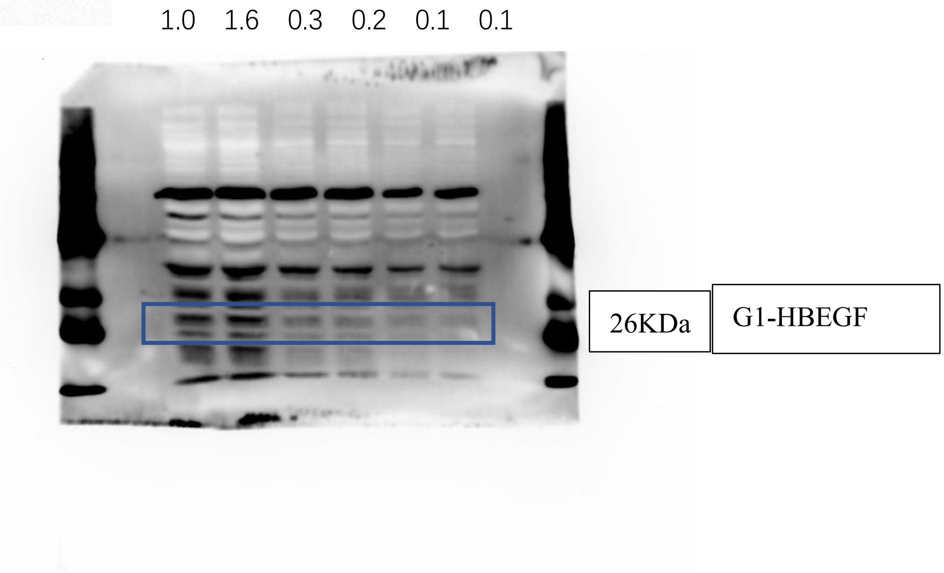
1.0 1.6 0.3 0.2 0.1 0.1
26KDa
G1-HBEGF

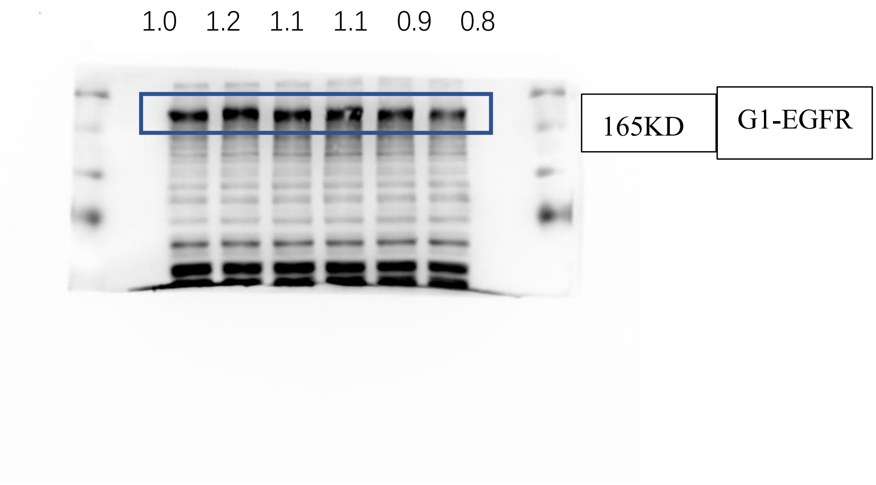
1.0 1.2 1.1 1.1 0.9 0.8
165KD
G1-EGFR

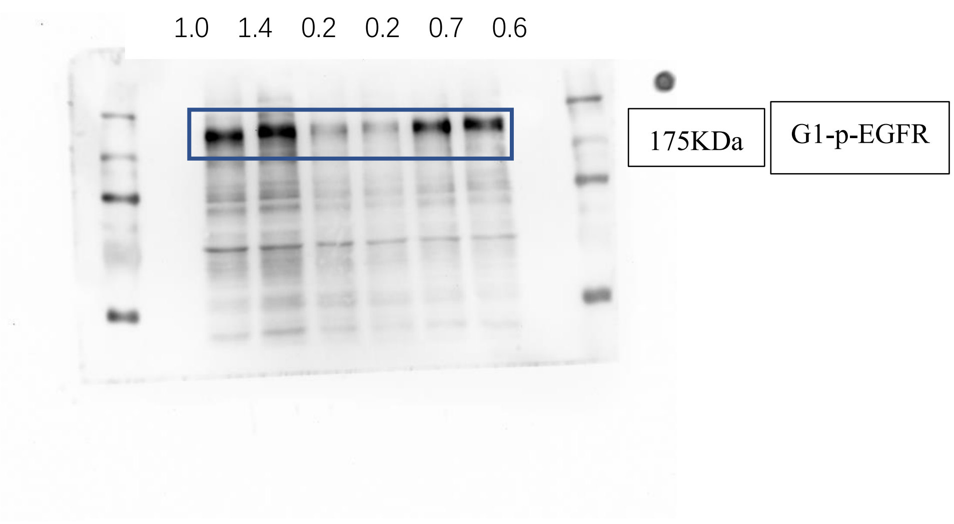

1.0 1.4 0.2 0.2 0.7 0.6
175KDa
G1-p-EGFR

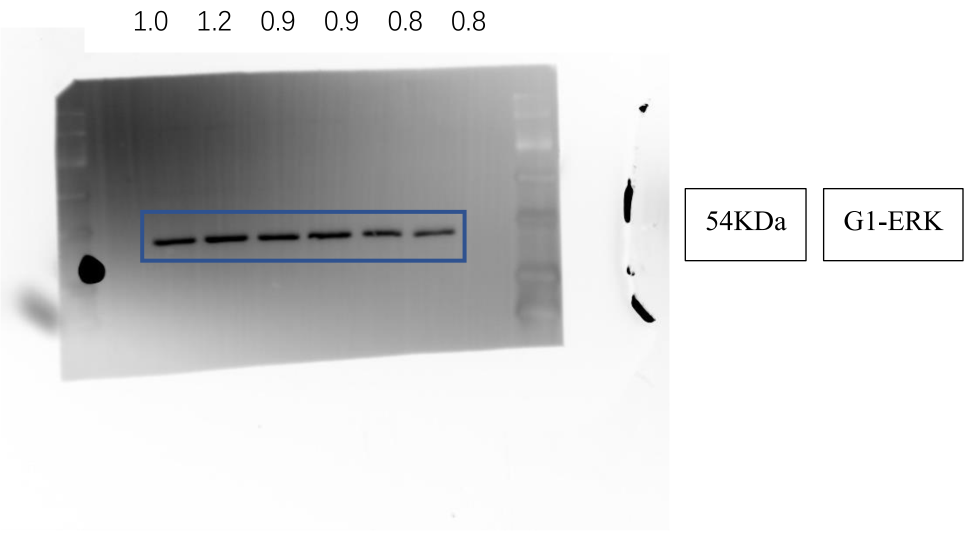

1.0 1.2 0.9 0.9 0.8 0.8
54KDa
G1-ERK

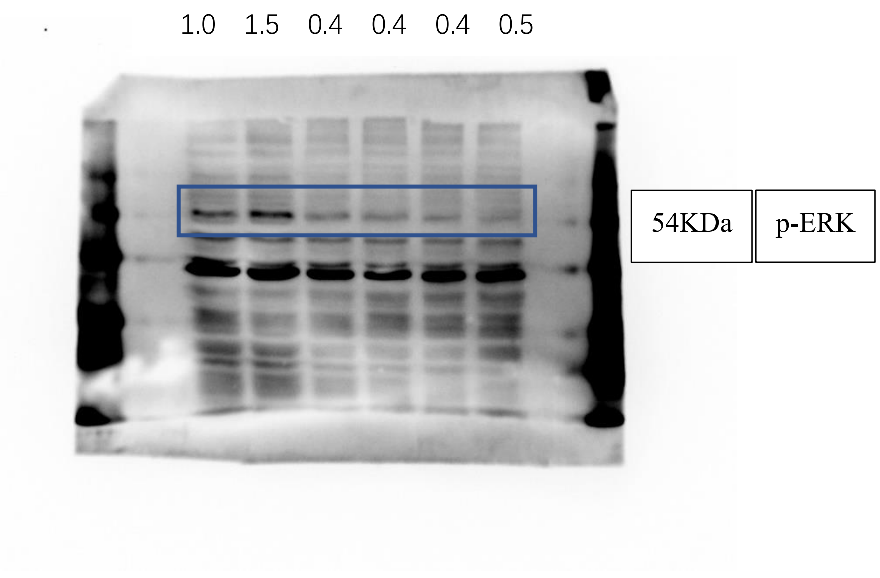

1.0 1.5 0.4 0.4 0.4 0.5
54KDa
p-ERK

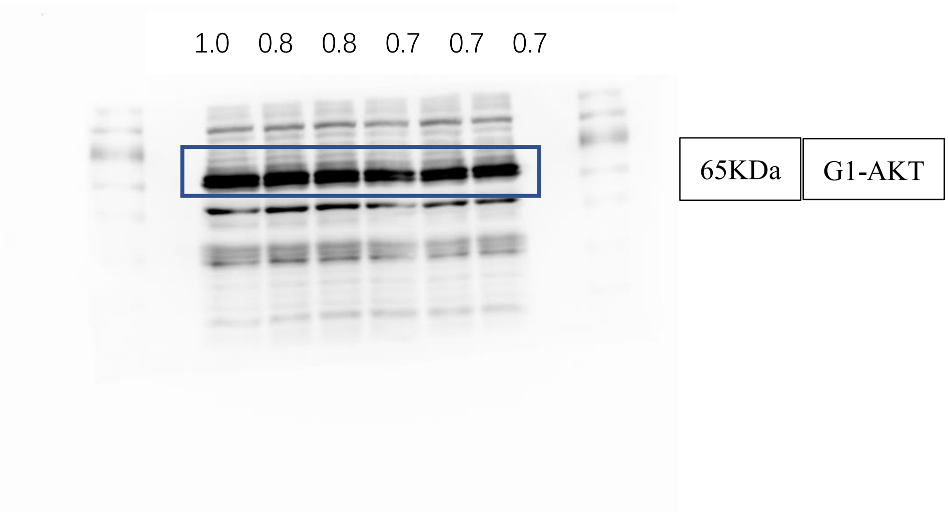
1.0 0.8 0.8 0.7 0.7 0.7
65KDa
G1-AKT

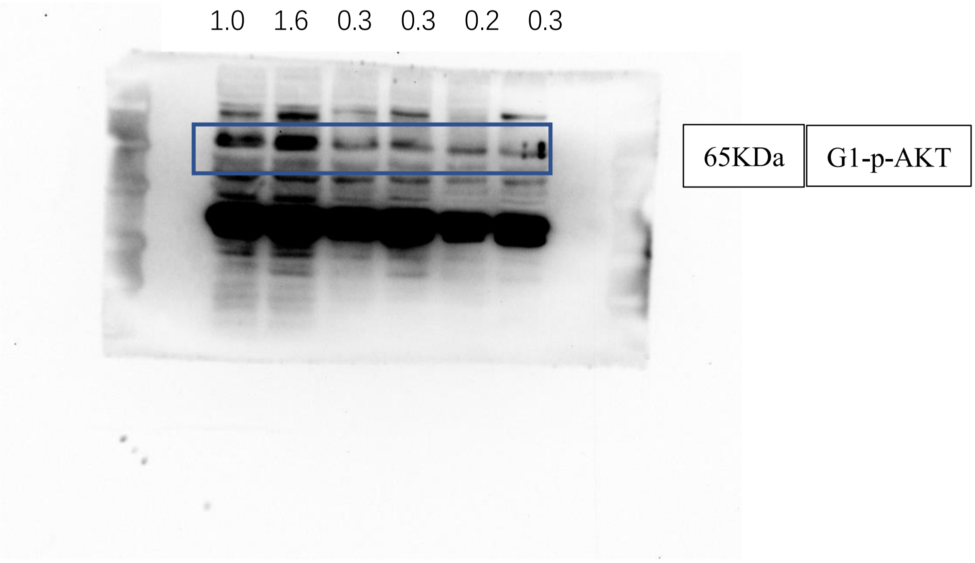
1.0 1.6 0.3 0.3 0.2 0.3
65KDa
G1-p-AKT

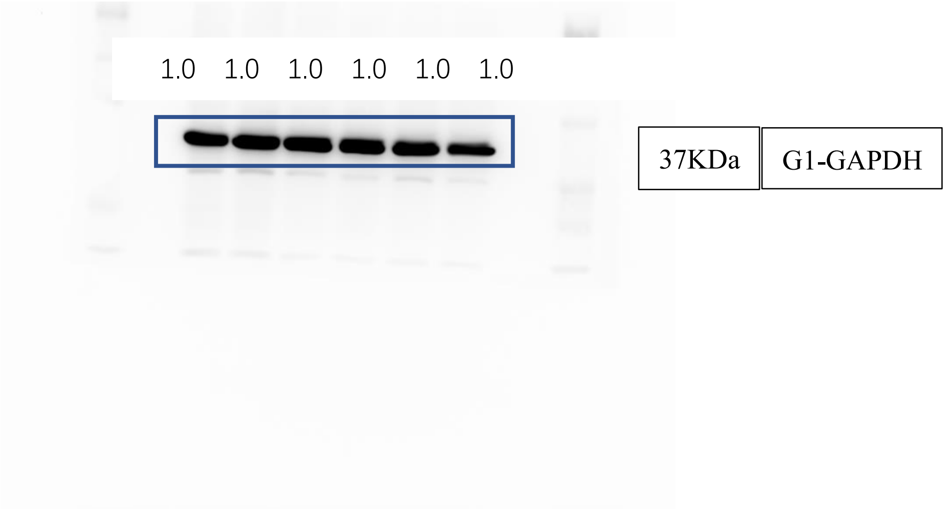
1.0 1.0 1.0 1.0 1.0 1.0
37KDa
G1-GAPDH

c

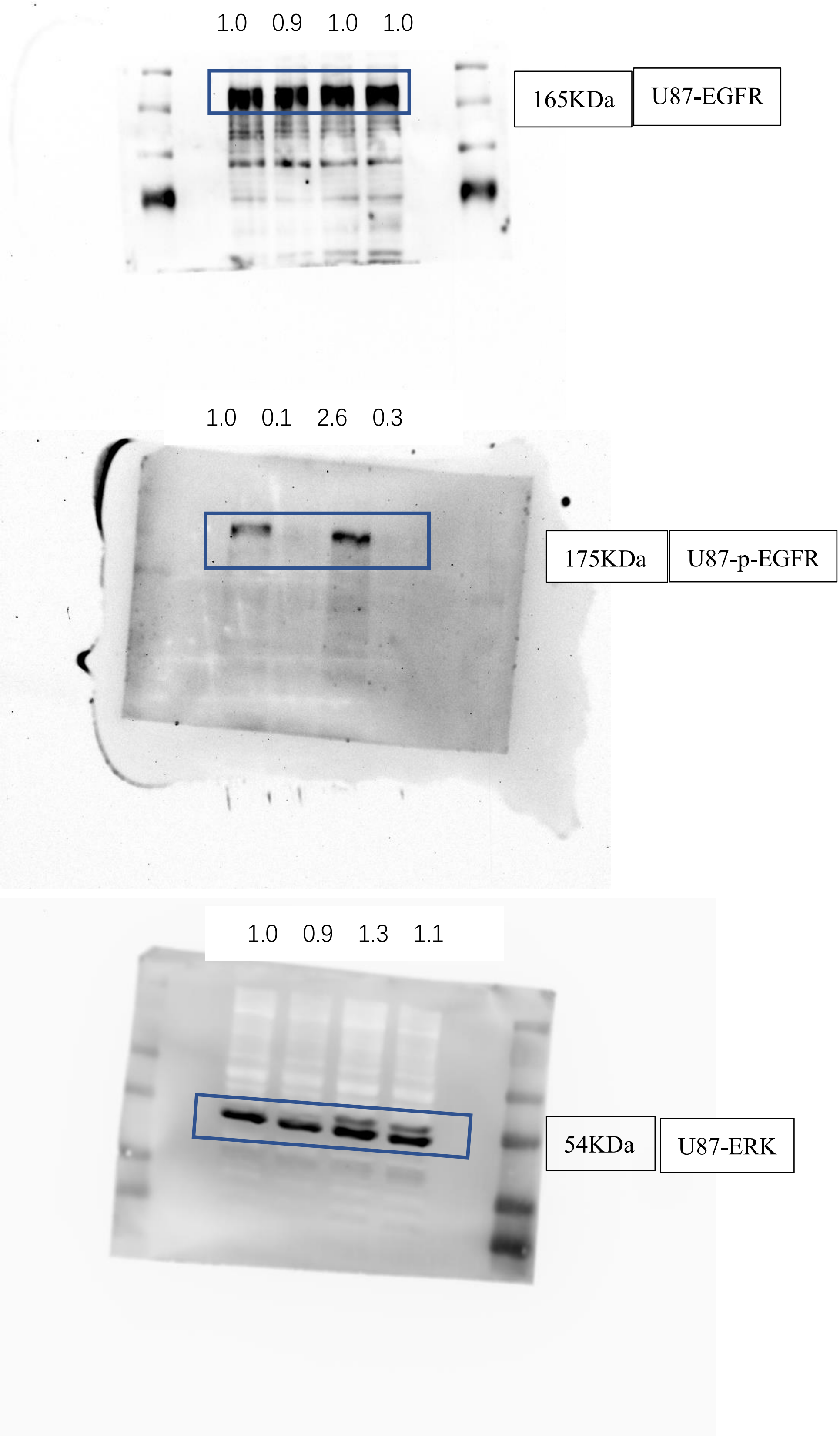
1.0 0.9 1.0 1.0
165KDa
U87-EGFR
1.0 0.1 2.6 0.3
175KDa
U87-p-EGFR
1.0 0.9 1.3 1.1
54KDa
U87-ERK

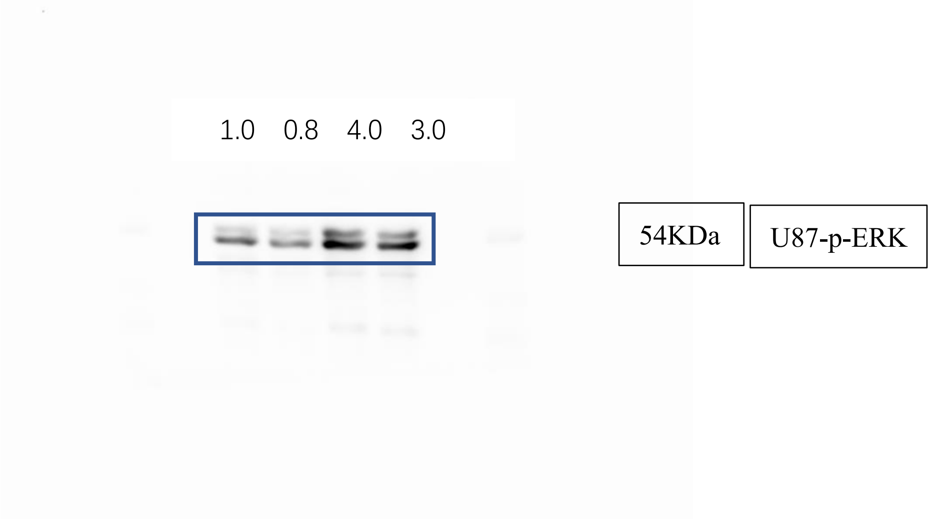
1.0 0.8 4.0 3.0
54KDa
U87-p-ERK

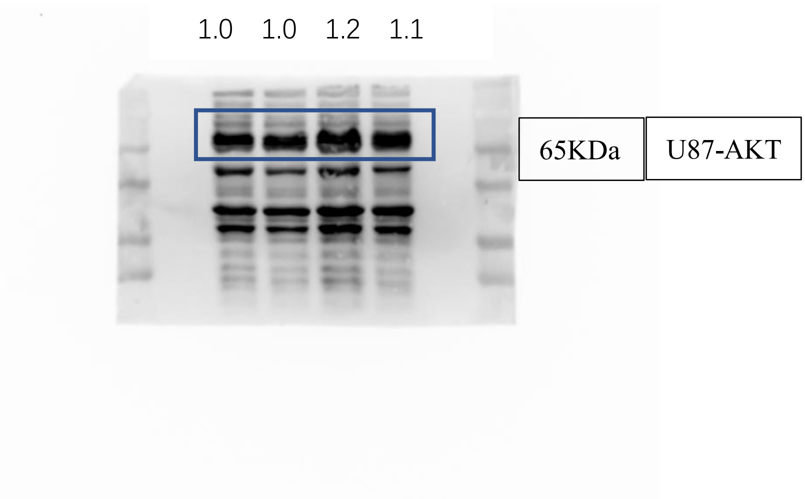
1.0 1.0 1.2 1.1
65KDa
U87-AKT

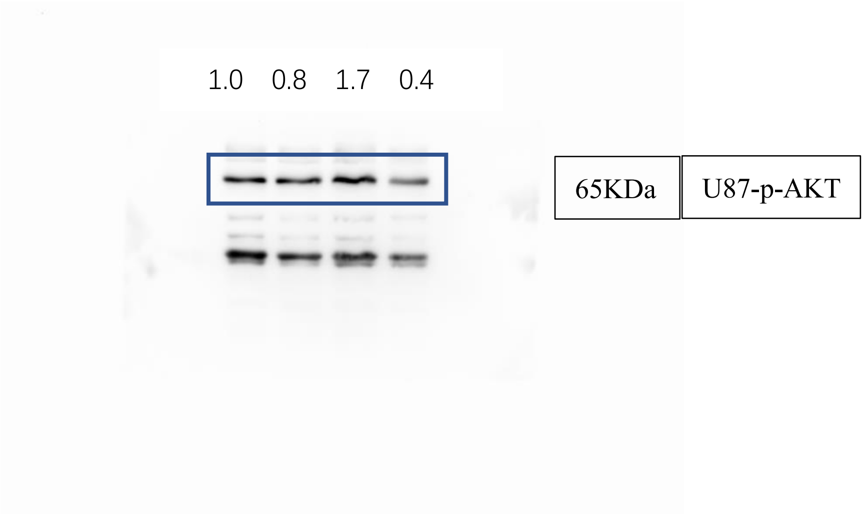
1.0 0.8 1.7 0.4
65KDa
U87-p-AKT

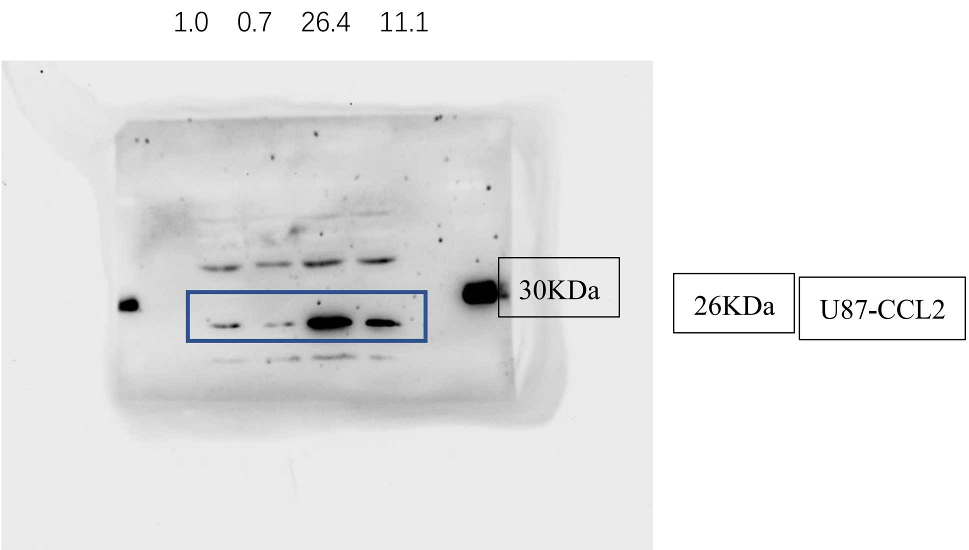
1.0 0.7 26.4 11.1
30KDa
26KDa
U87-CCL2

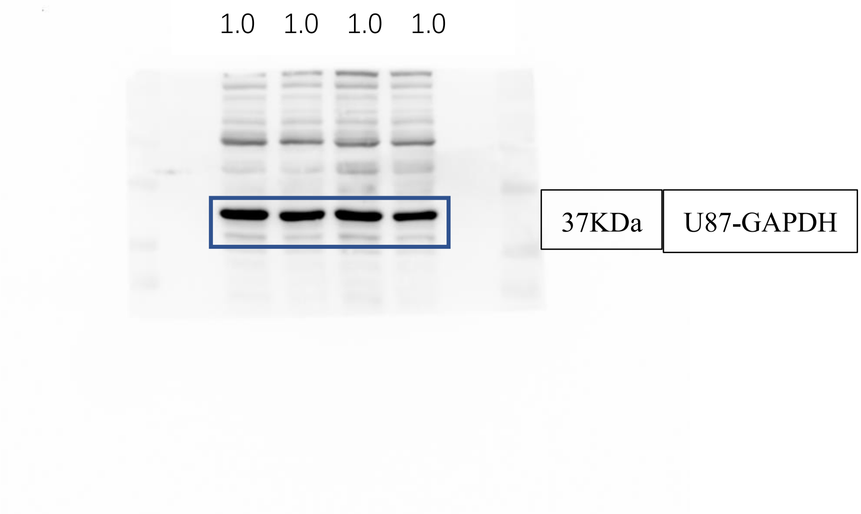
1.0 1.0 1.0 1.0
37KDa
U87-GAPDH

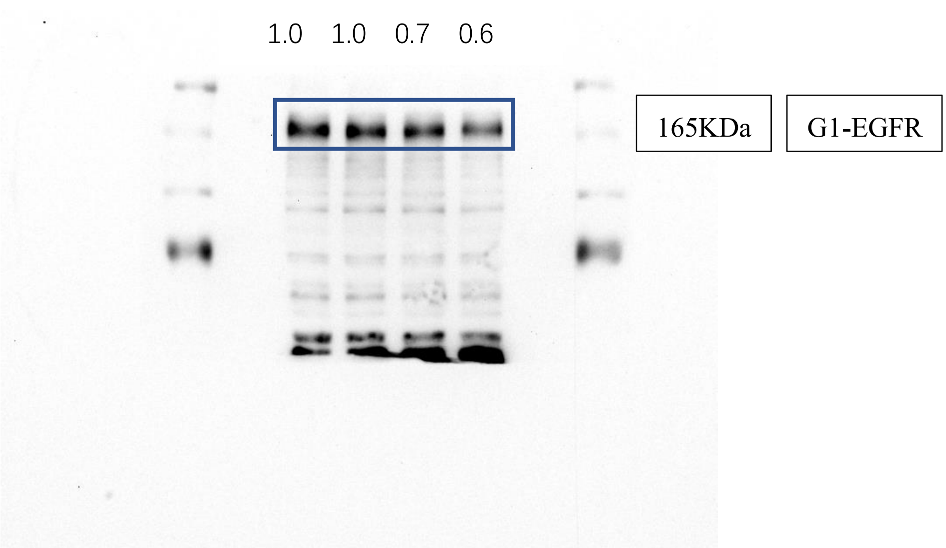
1.0 1.0 0.7 0.6
165KDa
G1-EGFR

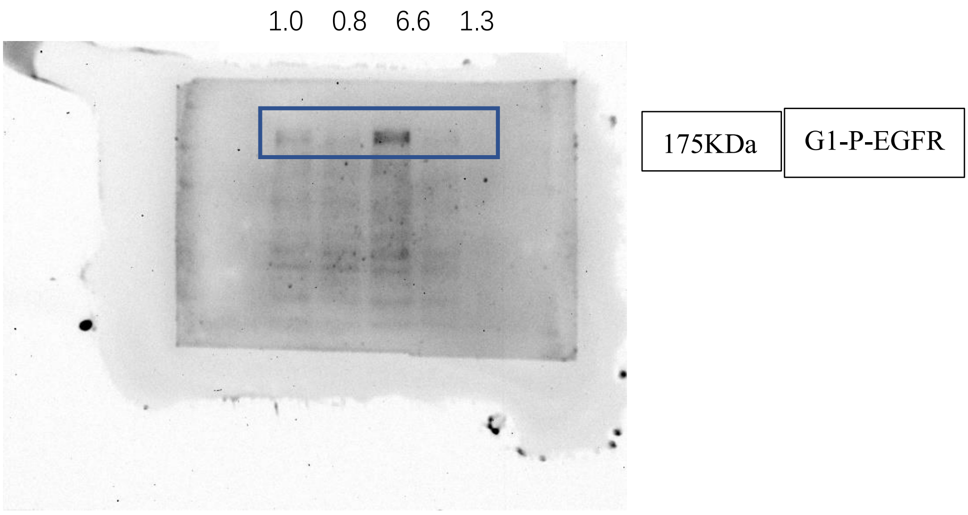
1.0 0.8 6.6 1.3
175KDa
G1-P-EGFR

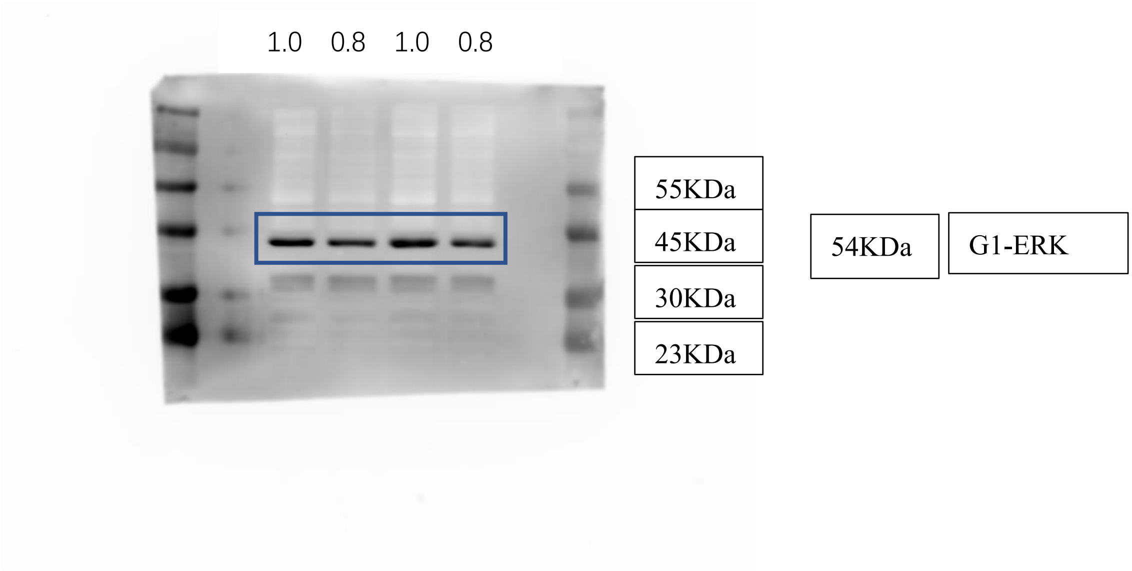
1.0 0.8 1.0 0.8
55KDa
45KDa
30KDa
23KDa
54KDa
G1-ERK

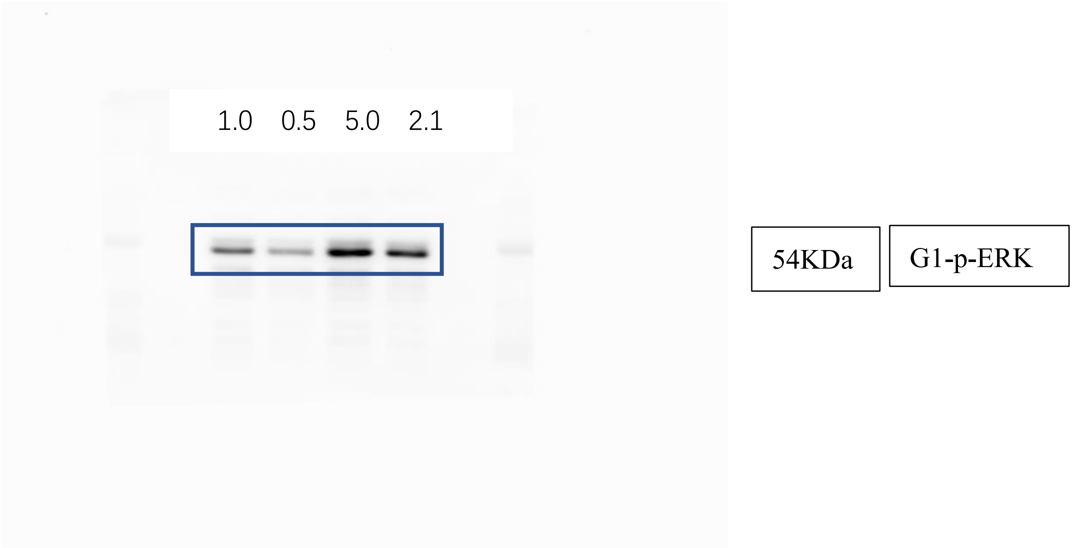
1.0 0.5 5.0 2.1
54KDa
G1-p-ERK

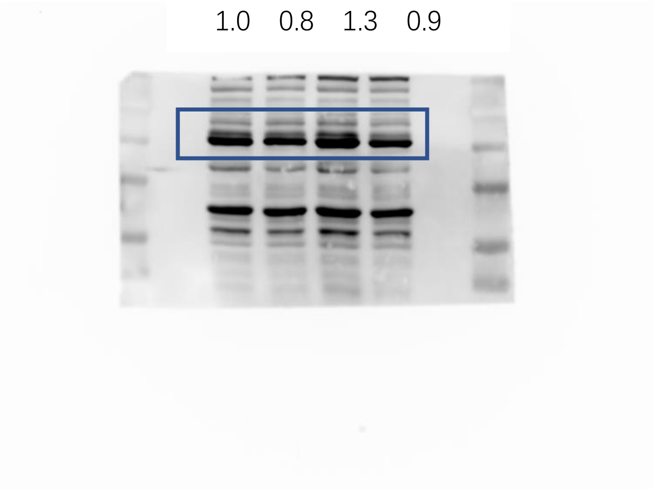

65KDa G1-akt

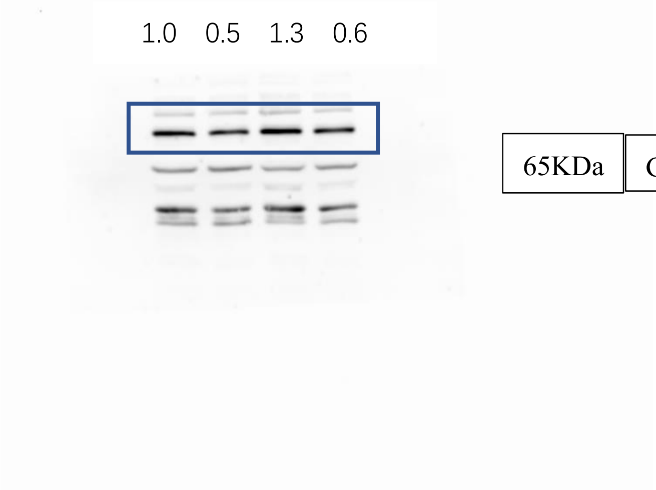

65KDa G1-p-akt

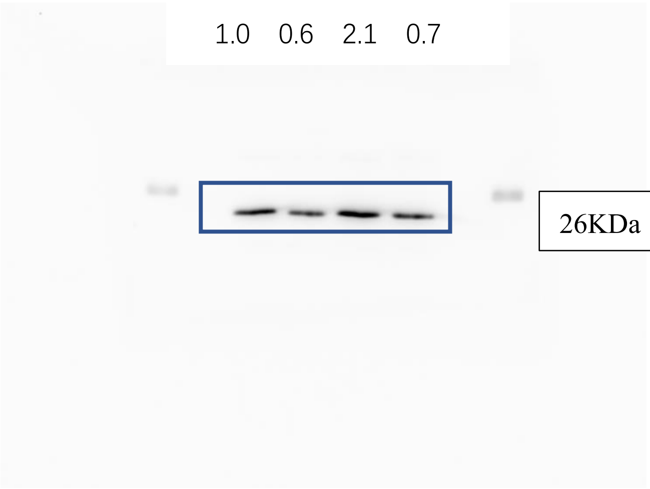

26KDa G1-CCL2

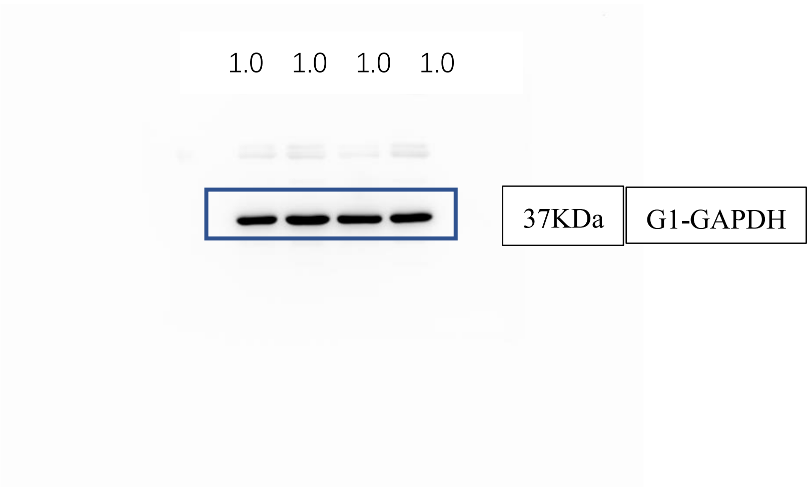
1.0 1.0 1.0 1.0
37KDa
G1-GAPDH

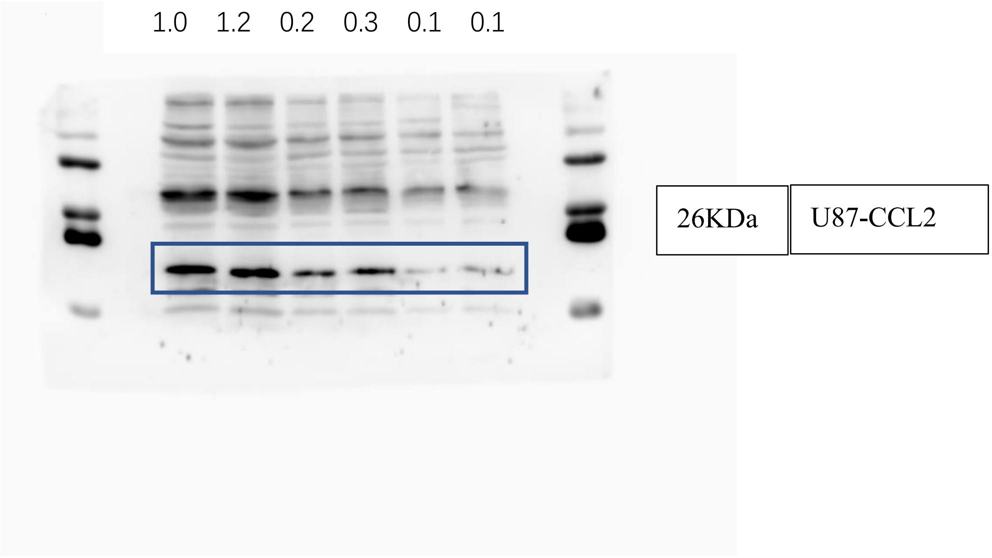
1.0 1.2 0.2 0.3 0.1 0.1
26KDa
U87-CCL2

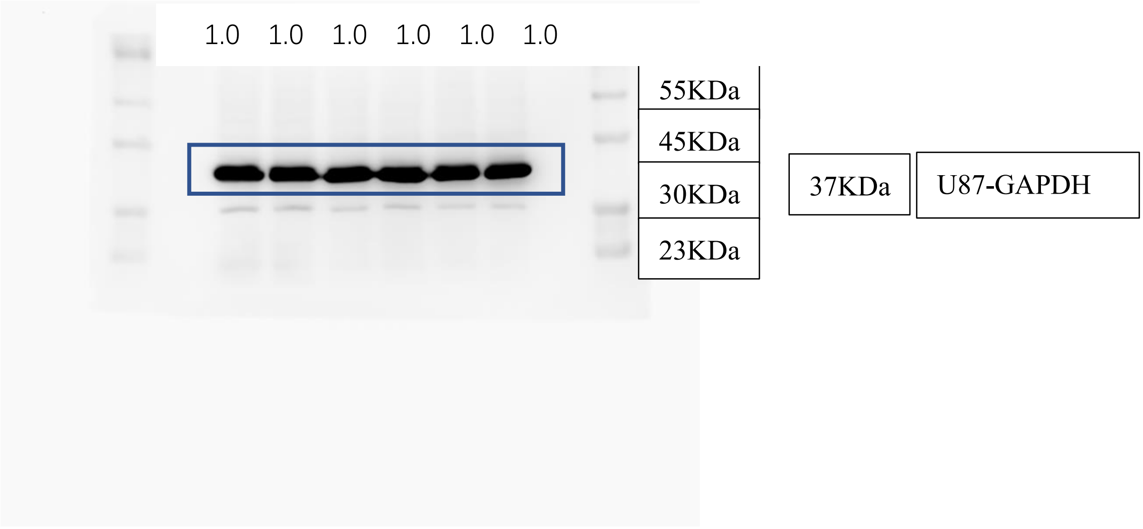
1.0 1.0 1.0 1.0 1.0 1.0
55KDa
45KDa
30KDa
23KDa
37KDa
U87-GAPDH

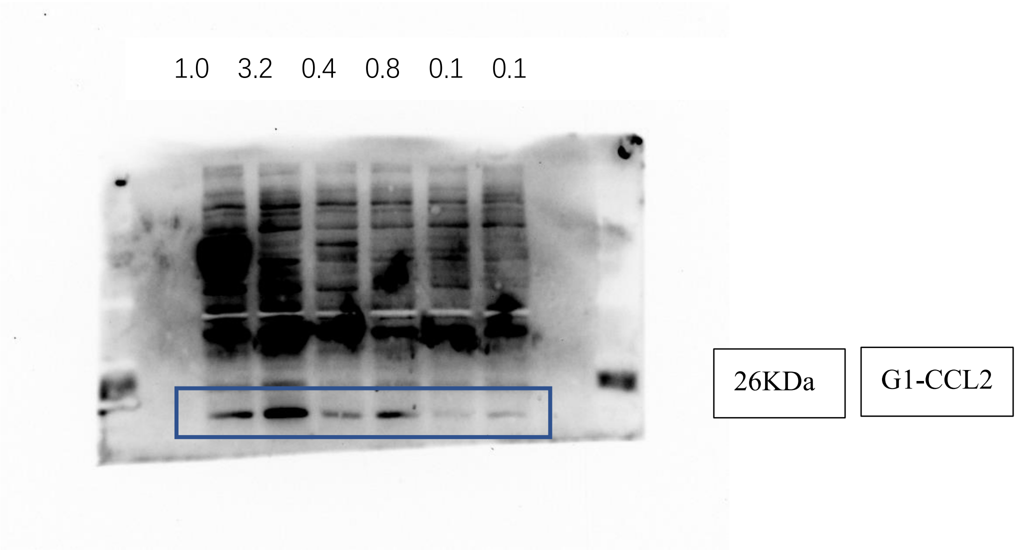

1.0 3.2 0.4 0.8 0.1 0.1
26KDa
G1-CCL2

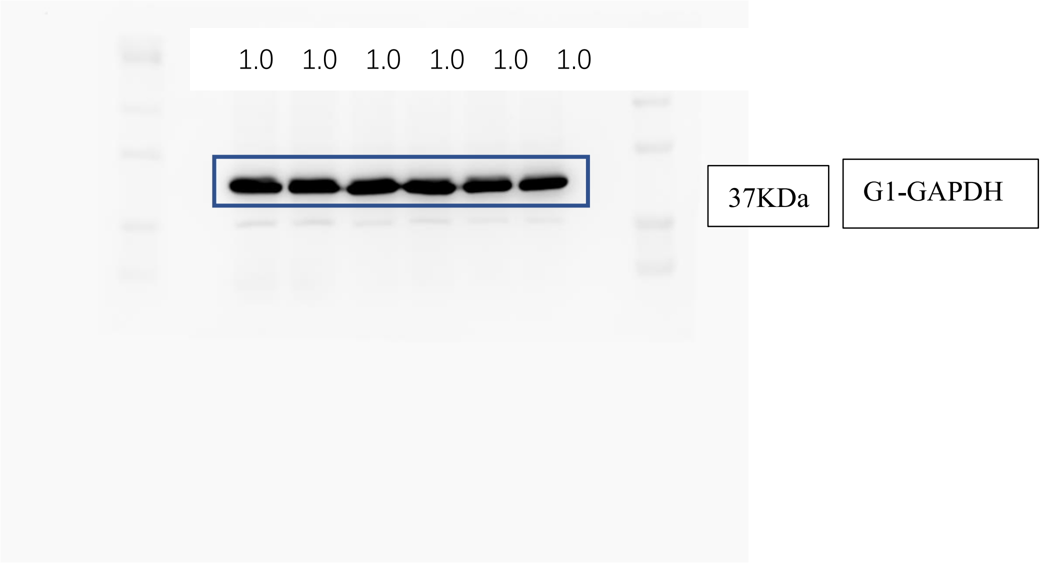

1.0 1.0 1.0 1.0 1.0 1.0
37KDa
G1-GAPDH
