# Supplementary material for: The GBM Tumor Microenvironment as a Modulator of Therapy Response: ADAM8 Causes Tumor Infiltration of Tams through HB-EGF/EGFR-Mediated CCL2 Expression and Overcomes TMZ Chemosensitization in Glioblastoma
Source: Cancers (Basel). 2022 Oct 7;14(19):4910. doi: 10.3390/cancers14194910 (PMC9561975; doi:10.3390/cancers14194910)
Supplement: Supplementary file 1 [file cancers-14-04910-s001.zip › Supplementary Data Tables S1 and S2.pdf]

Table S1. Antibodies and agents

Primary antibodies

Names	Company	Lot. No.
ADAM8	Proteintech (23778-1-AP)	00089073
EGFR	Proteintech (66455-1-2g)	10014314
AKT	Proteintech (60203-2-Ig)	10021367
p-AKT	Proteintech (66444-1-Ig)	10022023
ERK	Proteintech (11257-1-AP)	16443
CCL2	Proteintech (66272-1-Ig)	10011921
IBA-1	Abcam (ab5076)	GR3253755-1
CD206	Abcam (ab64693)	GR134288-1
GFAP	Abcam (ab53554)	GR119165-1
HB-EGF	Abclonal (A16365)	3507466001
p-ERK	CST (4370)	12
p-EGFR	CST (3777)	16
GAPDH	Enogene	E12-042

Secondary antibodies

Names	company	Lot. No.
Cy3-conjugated Goat anti-rabbit	Proteintech (SA00009-2)	20000328
488-conjugated Goat anti-mouse	Abbkine(A23210-1)	133404A
IFkine green Donkey anti-mouse	Abbkine(A24211-1)	133702A
488-conjugated Donkey anti-goat	Servicebio(GB25404)	CR2104104
Cy3-conjugated Donkey anti-rabbit	Servicebio(GB21403)	CR2105134
Peroxidase-conjugated goat anti-rabbit	Proteintech (SA00001-2)	20000003
Peroxidase-conjugated Goat anti-mouse	Proteintech (SA00001-1)	20000002

Main agents

Names	company	Lot. No.
Temozolomide	Selleck (s1237)	S123708
Erlotinib	Selleck (s1023)	S102305

ELISA kit

Human MCP-1 ELISA kit	Elabscience	E-EL-H6005

Table S2. Sequences of primer

Primers	Sequences
**ADAM8-F**	GATGATGCTGCCTGCGATTG
**ADAM8-R**	AAGGACGTAGCTCACCCTCT
**HBEGF-F**	GTCACTTTATCCTCCAAGCCAC
**HBEGF-R**	CTCAGCCCATGACACCTCTCT
**CCL2-F**	TCTCAAACTGAAGCTCGCACT
**CCL2-R**	ACACTTGCTGCTGGTGATTCT
**Mmp1-F**	AGAAAGAAGACAAAGGCAAGTTGA
**Mmp1-R**	CTCTTGGCAAATCTGGCGTG
**Mmp2-F**	GTCTGTGTTGTCCAGAGGCA
**Mmp2-R**	ATCACTAGGCCAGCTGGTTG
**Mmp7-F**	ACAATTGTCTCTGGACGGCA
**Mmp7-R**	CACATCTGGGCTTCTGCATT
**Mmp9-F**	TTGTGCTCTTCCCTGGAGAC
**Mmp9-R**	CCGAGTGTAACCATAGCGGT
**Mmp12-F**	CCTGAACAGCTCTACAAGCCT
**Mmp12-R**	TCCAGGGTAGATGTGTCCAGT
**Mmp14-F**	CAGTTCGCCGACTAAGCAGA
**Mmp14-R**	CGGGAGACATGGTCCGAGA
**ADAM8-F**	GATGATGCTGCCTGCGATTG
**ADAM8-R**	AAGGACGTAGCTCACCCTCT
**ADAM10-F**	TGCTGCTGTTAACCCGTGA
**ADAM10-R**	GATTCCCATACTGACCTCCCA
**ADAM12-F**	TGCCTTCTCTCACTAACGCT
**ADAM12-R**	GCTGAGCTCTTCTAGCCTTTC
**ADAM17-F**	GGCGGTAGAATCTTCCCAGT
**ADAM17-R**	ACCACGCTGGTCAGGAATAG
**ADAM19-F**	GCTCTGCTTGCTGGCGTTT
**ADAM19-R**	TTCTCGCCCCTCAGCCATTA
**GAPDH-F**	GGAAGCTTGTCATCAATGGAAATC
**GAPDH-R**	TGATGACCCTTTTGGCTCCC
