# Supplementary material for: The GBM Tumor Microenvironment as a Modulator of Therapy Response: ADAM8 Causes Tumor Infiltration of Tams through HB-EGF/EGFR-Mediated CCL2 Expression and Overcomes TMZ Chemosensitization in Glioblastoma
Source: Cancers (Basel). 2022 Oct 7;14(19):4910. doi: 10.3390/cancers14194910 (PMC9561975; doi:10.3390/cancers14194910)
Supplement: Supplementary file 1 [file cancers-14-04910-s001.zip › Supplementary Figures S1-S4.pdf]

Supplementary Figures legend

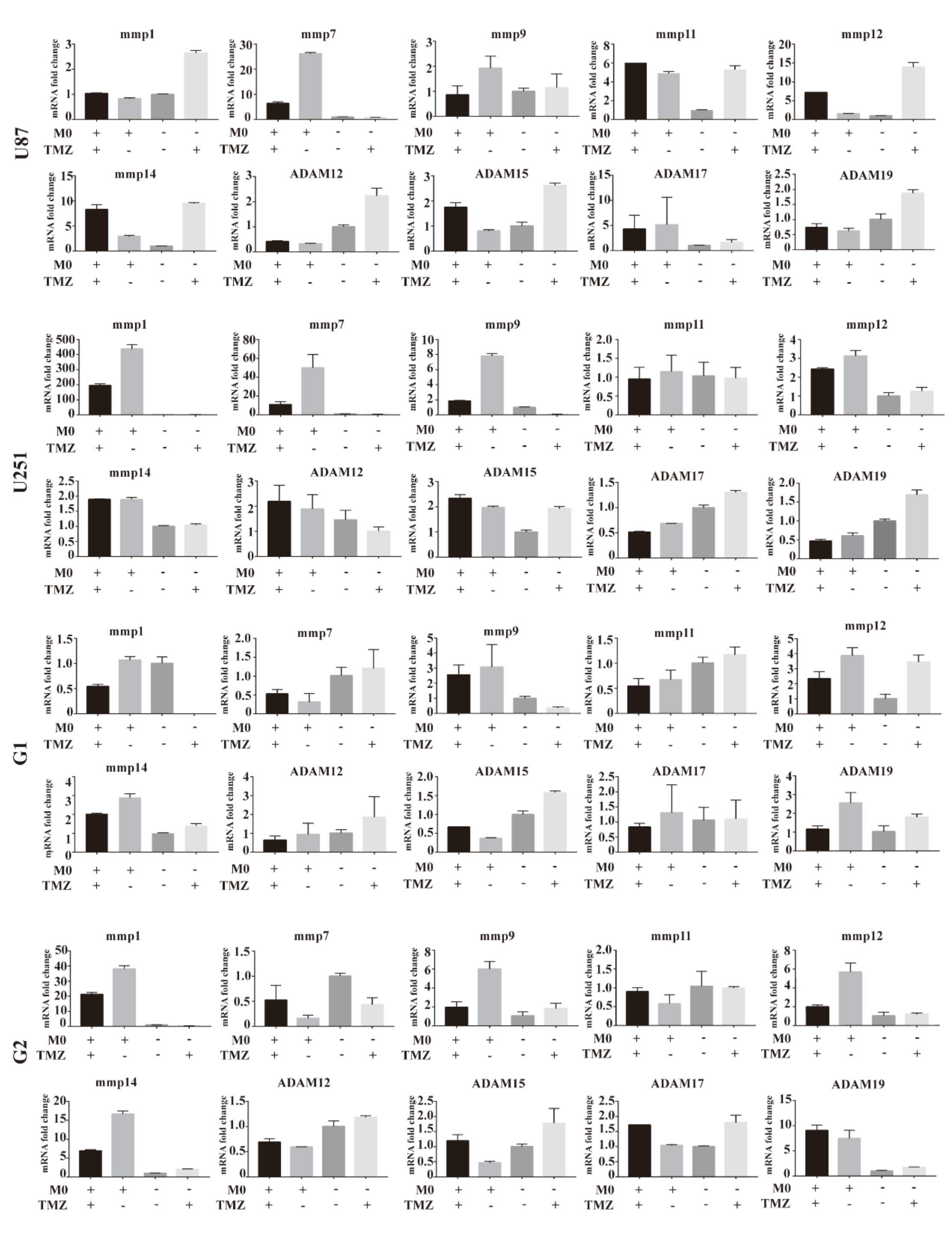

Figure S1. qPCR screening of MMPs and ADAMs genes. qPCR was used to screen the MMPs (mmp1, mmp7, mmp9, mmp11, mmp12 and mmp14) and ADAMs (ADAM8, ADAM12, ADAM15, ADAM17, and ADAM19) genes in GBM cell lines

(U87MG and U251MG) as well as primary cells (G1 and G2) under TMZ treatment (500ng/ml) and co-culture with THP-1 derived macrophages for 3 days.

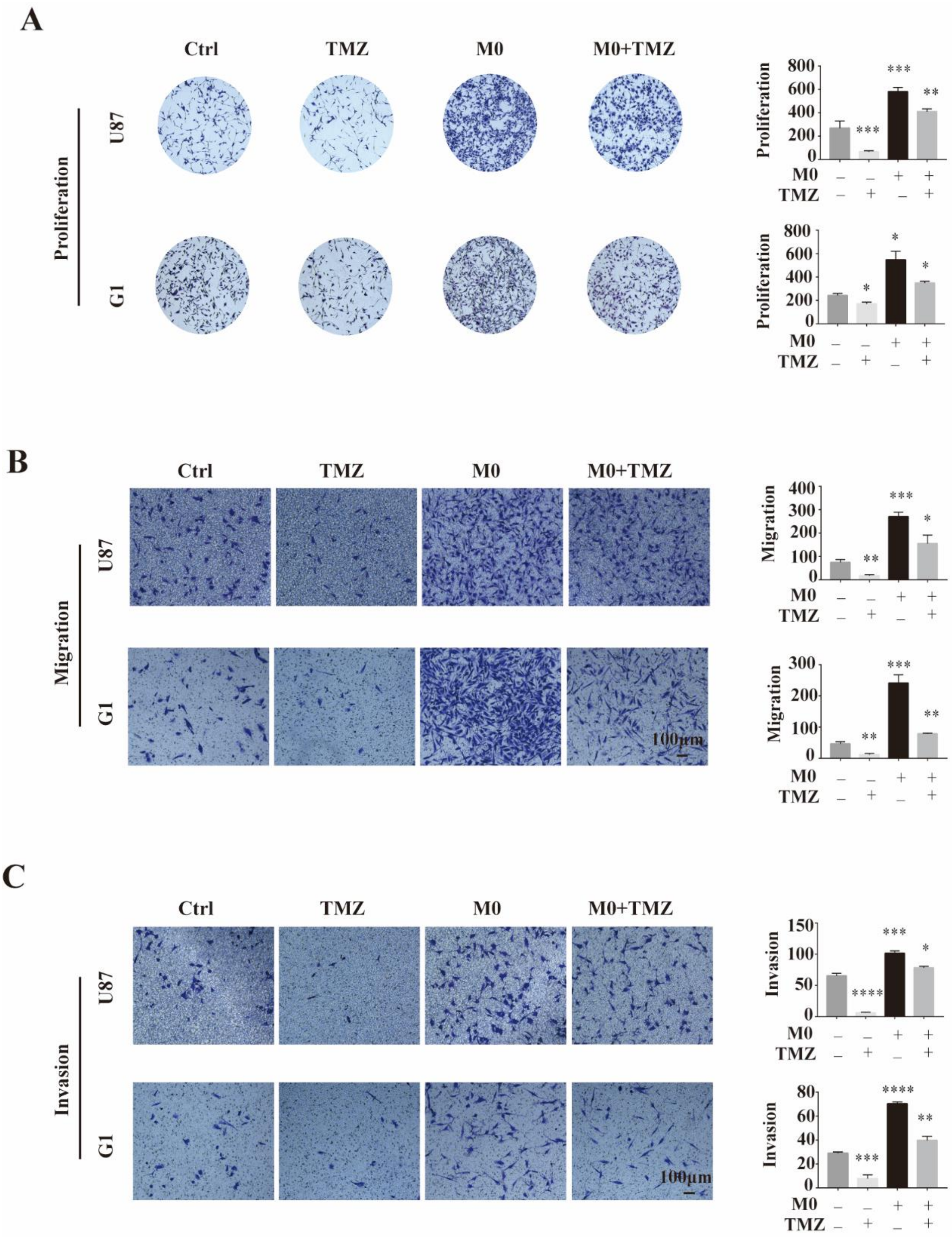

Figure S2. Macrophages promoted GBM cells chemoresistance. A Co-cultures of GBM cells with macrophages were performed in the presence of TMZ. GBM cells in the lower chamber of each group were stained and visualized under the microscope.

The number of GBM cells was measured by ImageJ and the statistical results of proliferation assay were shown in the bar graph. **B, C** Cell migration and invasion assay was performed in GBM cells when treated differently. All experiments were carried out in triplicate, and data are presented as mean ± SD. scale bar = 100um. *$p < 0.05$, **$p < 0.01$, ***$p < 0.001$ **** $p < 0.0001$.

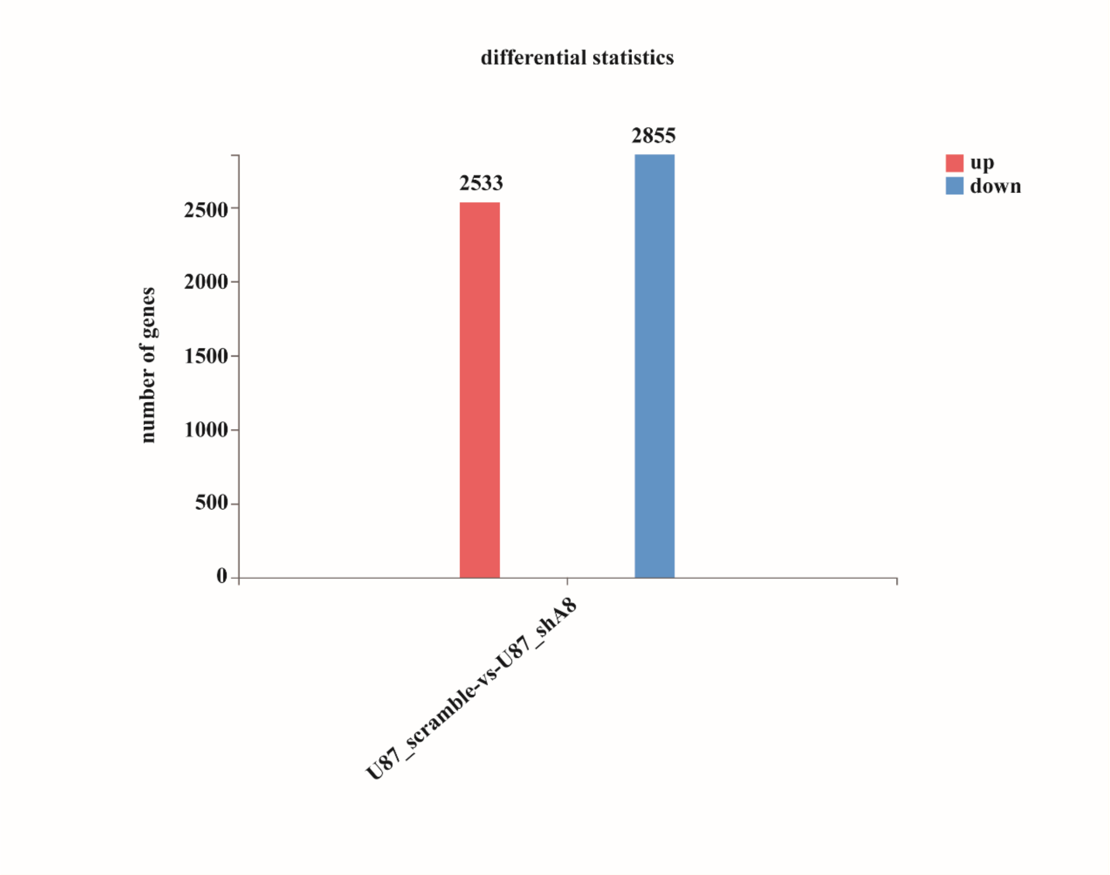

Figure S3. Differential statistics of genes. RNA sequencing was performed on U87MG_shA8, and U87MG_scramble cells. The bar graph represented Differential statistics of genes.

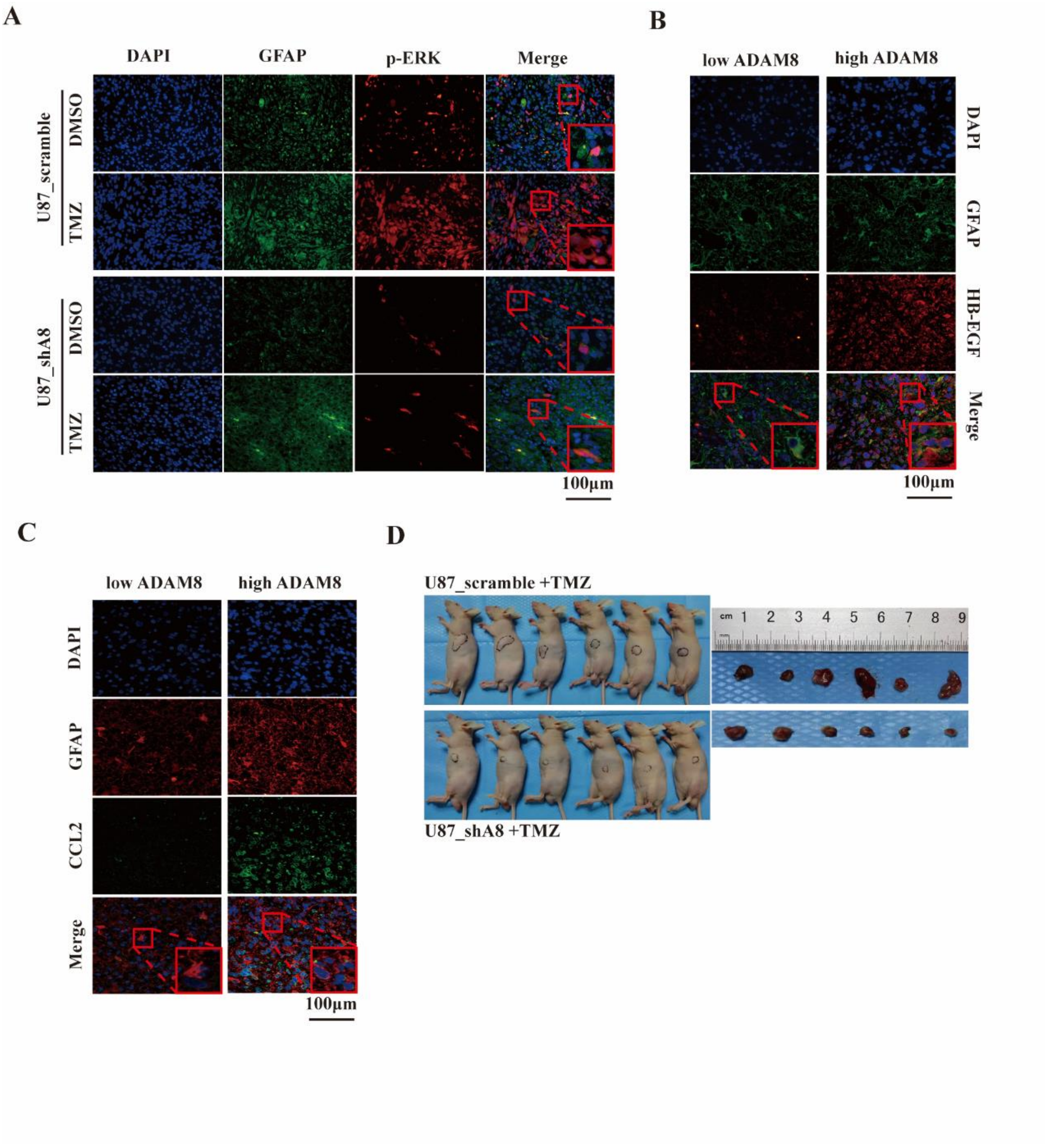

Figure S4. ADAM8 regulate HB-EGF/EGFR mediated CCL2 expression. A Immunofluorescence staining of p-ERK of xenograft tissue sections. **B, C** Immunofluorescence staining of HB-EGF and CCL2 in our collected GBM cohorts, respectively. **D** Extracted tumor from subcutaneous U87MG xenograft models were shown in the picture.
